# Electroporation enables the efficient mRNA delivery into the mouse zygotes and facilitates CRISPR/Cas9-based genome editing

**DOI:** 10.1038/srep11315

**Published:** 2015-06-11

**Authors:** Masakazu Hashimoto, Tatsuya Takemoto

**Affiliations:** 1 Division of Developmental Biology, Graduate School of Medicine, Chiba University, Inohana 1-8-1, Chuo-ku, Chiba 260-8670, Japan; 2 Division of Embryology, Fujii Memorial Institute of Medical Sciences, Tokushima University, 3-18-15 kuramoto-cho, Tokushima 770-8503, Japan

## Abstract

Recent use of the CRISPR/Cas9 system has dramatically reduced the time required to produce mutant mice, but the involvement of a time-consuming microinjection step still hampers its application for high-throughput genetic analysis. Here we developed a simple, highly efficient, and large-scale genome editing method, in which the RNAs for the CRISPR/Cas9 system are electroporated into zygotes rather than microinjected. We used this method to perform single-stranded oligodeoxynucleotide (ssODN)-mediated knock-in in mouse embryos. This method facilitates large-scale genetic analysis in the mouse.

The mouse is a widely used mammalian model organism. Since the completion of the mouse genome sequence, much research has focused on the functional characterization of each gene in various biological processes, including development and disease. The production of genetically modified animals is essential for elucidating the roles of genes, but the traditional method, which involves homologous recombination in embryonic stem cells and the production of chimeric mice, is costly and time-consuming. However, new genome editing approaches have dramatically changed the ways genetically modified mice are generated^1^. The introduction of sequence-specific artificial nucleases into zygotes generates DNA double-strand breaks (DSBs). The DSBs activate two endogenous repair processes: error-prone non-homologous end joining (NHEJ), which introduces insertions or deletions (indels), and homology-directed repair (HDR), which involves homologous recombination and can introduce exogenous sequences. Among efficient genome editing methods, the CRISPR/Cas9 system is the simplest for generating mice carrying a modified genome^2^,^3^,^4^. In most reports using this system, *Cas9* mRNA and guide RNA (gRNA, a fusion of CRISPR crRNA and trans-activating CRISPR RNA), or plasmid(s) expressing these RNAs, are introduced into the zygote by microinjection^5^,^6^.

Although the CRISPR/Cas9 system simplifies the procedure for producing mutants, the microinjection of DNAs/RNAs into zygotes requires special skill and is too time-consuming to produce mutant mice on a large scale (e.g., by injecting hundreds of zygotes). Therefore, to simplify the process, we sought to examine whether electroporation could be used to introduce the RNAs into zygotes. Electroporation was previously applied to mouse zygotes^7^, but the process required that the zona pellucida be removed or thinned by treatment with acid Tyrode’s solution, which is toxic to embryos, and only short RNAs (less than 1 kb) were introduced. Recently, a mutant rat was successfully produced by electroporating *Cas9* mRNA and gRNA into rat zygotes with an intact zona pellucida, but the efficiency of genome editing was quite low (less than 9%), and a very high concentration of *Cas9* mRNA (1000–2000 ng/μl) was required^8^.

To achieve efficient genome editing in mouse embryos using electroporation, we started by optimizing the electroporation conditions for introducing mRNA into fertilized mouse eggs with an intact zona pellucida. We set up the electroporation system as shown in Fig. 1a. A custom-made electroporation chamber, in which two platinum block electrodes (10-mm long, 3-mm wide, and 0.5-mm thick) were situated with a 1-mm gap between them (Fig. 1b,c), was placed under a stereoscopic microscope and connected to an electric pulse generator. The chamber held 5 μl of RNA solution between the electrodes, and allowed approximately 40–50 embryos to be electroporated simultaneously (Fig. 1c,d).

To optimize the conditions, we used 400 ng/μl mCherry mRNA, and evaluated both the efficiency of mRNA introduction, by monitoring the fluorescence intensity of mCherry, and the rate of embryo survival to the blastocyst stage. We first tested a range of electroporation voltages (10 V–50 V) while keeping the duration and number of pulses fixed at 3 msec and five repeats, respectively (Fig. 1e). We found that 30 V was the most efficient condition for mRNA introduction (Fig. 1f). That is, the survival rate was lower at 40 V or above, and the amount of mRNA introduced was lower at 20 V or less. Next, fixing the voltage and duration at 30 V and 3 msec, respectively, we changed the number of pulses (x3, x5, x7, x9, and x11). Although the intensity of the mCherry increased with the number of repeats, the survival rate of the electroporated embryos significantly decreased at nine repeats (Fig. 1g). Therefore, we concluded that seven repeats of 30-V, 3-msec pulses are the optimal conditions for introducing mRNA into mouse zygotes by electroporation.

We next examined whether the optimized conditions were conducive to CRISPR/Cas9-mediated genome editing. We targeted the *Fgf10* gene, because *Fgf10* homozygous mutant embryos have an easily detectable limbless phenotype^9^. Using electroporation, we introduced *Cas9* mRNA and a gRNA targeting *Fgf10* (#563), which was previously transferred into eggs by the microinjection method and elicited the limbless phenotype^6^ (Fig. 2a). The eggs were allowed to develop to the two-cell stage and then transferred into pseudopregnant females. The transferred embryos were subsequently dissected on embryonic day (E) 15 or E16, and their phenotypes were analyzed. We performed electroporations using four different concentrations of *Cas9* mRNA and gRNA. At all of the concentrations examined, the survival rate of the electroporated zygotes that developed to the two-cell stage was much higher (94–95%; Supplementary Table S1) than when using the microinjection method (34–35% in ref. 6).

Depending on the limb-development defects observed at E15 or E16, the embryos were classified into three phenotypes: type I embryos had no limbs (*Fgf10* gene knockout phenotype), type II embryos showed various defects in limb morphology (e.g., hindlimb deficiency or truncated fore- and hindlimbs), and type III embryos appeared normal (Fig. 2b). Considering that mutant mice heterozygous for the *Fgf10* gene have normal limbs, type II embryos were expected to be mosaic mutant embryos composed of cells carrying a bi-allelic disruption of the *Fgf10* gene and cells carrying either the wild-type or a mono-allelic disruption of the *Fgf10* gene. When we used 400 ng/μl *Cas9* mRNA and 200 ng/μl gRNA for the electroporation, 87% of the embryos completely lacked both fore- and hindlimbs, as expected for the *Fgf10* homozygous mutant phenotype (type I) (Fig. 2c); 10% of the embryos displayed various other limb defects (type II); and only 3% of the embryos appeared normal (type III). Sequencing of the genomic regions flanking the target sequences of type I embryos (ten clones each for four randomly selected embryos) revealed that all of the sequenced clones carried indels or mutations, and no wild-type sequence was detected (Supplementary Fig. S1 and Supplementary Table S2). These results indicate that both alleles of the *Fgf10* gene were disrupted.

We next examined the effect of the *Cas9* mRNA and gRNA concentrations on the efficiency of genome editing. At 200 ng/μl *Cas9* mRNA and 100 ng/μl gRNA, 73% of the embryos had no limbs, while at 100 ng/μl *Cas9* mRNA and 50 ng/μl gRNA, 32% displayed the limbless phenotype (Fig. 2b,c and Supplementary Table S1). At 50 ng/μl *Cas9* mRNA and 25 ng/μl gRNA, most of the embryos appeared normal. Sequencing revealed that embryos exhibiting the type I phenotype carried indels in all of their clones, whereas most of the clones from type III embryos had the wild-type sequence but some carried indels (Fig. 2b,c, Supplementary Fig. S1 and Supplementary Table S2). All of the clones from each electroporated embryo displayed two to five types of indels, which is comparable to the number in microinjected embryos. These results clearly demonstrated that electroporation was effective for achieving highly efficient genome editing by the CRISPR/Cas9 system.

The single-stranded oligodeoxynucleotide (ssODN)-induced generation of HDR-mediated insertions is another useful application of the genome-editing method. Therefore, we next examined whether electroporation could deliver ssODNs and generate HDR-mediated knock-in alleles in mice. We electroporated 400 ng/μl ssODN harboring loxP and EcoRI recognition sequences (37 bases) flanked by 40-base homologous arms (117 total bases), together with 200 ng/μl *Cas9* mRNA and 100 ng/μl gRNA targeting the *mCherry* gene, into eggs carrying a *Histone H2b (H2b)-mCherry* gene inserted into the *ROSA26* locus by conventional gene targeting (Fig. 3a)^10^. The allele replaced by the ssODN would be functionally null due to the introduction of a stop codon in the loxP sequence, leading to the disappearance of nuclear mCherry fluorescence. Replacement by the ssODN was screened by Restriction Fragment Length Polymorphism (RFLP) analysis after EcoRI digestion.

Confirming the high efficiency of genome editing, all of the electroporated embryos (11/11) exhibited a loss of mCherry fluorescence (Fig. 3b). Among them, four embryos were positive for EcoRI digestion (Fig. 3c). Sequencing revealed that all four embryos carried the HDR-mediated replaced allele (Supplementary Table S3). Of the four embryos with the replaced allele, three (#6, 8, and 9 in Supplementary Table S3) carried one to three types of alleles with indels, in addition to the replaced allele. The remaining embryo (#3 in Supplementary Table S3) carried only the replaced allele, indicating that all of the cells carried an allele with the HDR-mediated replacement sequence. We also achieved the HDR-mediated knock-in of an EcoRV site into the mCherry gene, and of an XbaI site into the *Fgf10* gene (Supplementary Fig. S2 and Supplementary Table S4). These results indicate that our electroporation method can also be used for the efficient production of HDR-mediated knock-in alleles in mice.

We next investigated whether the mutation obtained by genome editing was inherited by the next generation. We electroporated 200 ng/μl *Cas9* mRNA and 100 ng/μl gRNA targeting the *mCherry* gene into eggs carrying the *H2b-mCherry* gene in the *ROSA26* locus (Fig. 3a)^10^, and generated twenty-five F0 mice that carried the disrupted *mCherry* gene. Three F0 males were then intercrossed with wild-type female mice, and the resulting embryos or pups were tested for germline transmission of the disrupted *mCherry* gene by analyzing the mCherry fluorescence and the *mCherry* gene sequence. We found that all of the F1 embryos and pups were negative for mCherry fluorescence and carried the disrupted *mCherry* gene (Supplementary Table S5), indicating that the mutations introduced by genome editing were inherited by the next generation.

In conclusion, we established electroporation conditions that enable CRISPR/Cas9-based genome editing to be performed in mice at high efficiency and with a high rate of survival. Because electroporation does not require microinjection skills and can be used to treat 40–50 embryos simultaneously, this method produces mutant mice much more easily and quickly than previously reported. With this method, the high-throughput and/or large-scale production of mutant mice and/or mouse embryos becomes feasible for many laboratories. Applications of this method include the screening of developmentally important genes by analyzing F0 phenotypes.

## Methods

### Animals

All animal care and experiments were carried out in accordance with the Guidelines for Animal Experiments of Tokushima University and of Chiba University, and were approved by Institutional Animal Care and Use Committee of Tokushima University (Approval number: 14022) and the Animal Care and Use Committee of Chiba University (Approval number: 26-111, 26-304).

### mRNA and ssODN preparation

pCS2-mCherry was kindly provided by Dr. N. Kinoshita (NIBB). The hCas9 plasmid (pX330) was purchased from Addgene (Cambridge, MA). The hCas9 gene was excised from pX330, then placed downstream of the SP6 promoter in the pSP64 vector (Promega) (pSP64-hCas9) and used for RNA synthesis. pCS2-mCherry and pSP64-hCas9 were linearized by digestion with NotI and SalI, respectively, and used as templates for *mCherry* and *hCas9* mRNA synthesis using an *in vitro* RNA transcription kit (mMESSAGE mMACHINE SP6 Transcription Kit, Ambion, Austin, TX), according to the manufacturer’s instructions.

A pair of oligos targeting *Fgf10* or *mCherry* was annealed and inserted into the BsaI site of the pDR274 vector (Addgene). The sequences of the oligos were as follows: Fgf10 (5’-GGAGAGGACAAAAAACAAGA-3’) and mCherry (5’- GGCCACGAGTTCGAGATCGAGGG -3’). After digestion with DraI, gRNAs were synthesized using the MEGAshortscript T7 Transcription Kit (Ambion, Austin, TX). The synthesized mRNAs and gRNAs were purified by phenol-chloroform-isoamylalcohol extraction and isopropanol precipitation. The precipitated RNA was dissolved in Opti-MEM I (Life Technologies) at 2–4 μg/μl, and stored at –20 °C until use. RNAs were quantified by absorption spectroscopy and agarose gel electrophoresis. The ssODNs were purchased from Sigma in dry form, dissolved in Opti-MEM I to 1 μg/μl, and stored at –20 °C until use.

### Mice, and egg and embryo collection and transfer

We used ICR and B6D2F1 (C57BL/6 x DBA2 F1) female mice in this study. The ICR strain was mainly used for determining the optimal conditions for electroporation, and the B6D2F1 strain was used for genome editing.

Fertilized eggs were collected from the oviducts of E0.5 (12 hours after the midpoint of the day of vaginal plug) ICR or B6D2F1 females naturally intercrossed with males of the same strain. The covering cumulus cells were removed by incubating in 1% hyaluronidase/M2 medium (Sigma). For the genome editing experiments targeting *H2b-mCherry*, the eggs were obtained from B6D2F1 females intercrossed with R26-H2b-mCherry males. The collected eggs were pre-cultured in mWM medium until electroporation.

### Electroporation

**Figure 1:**
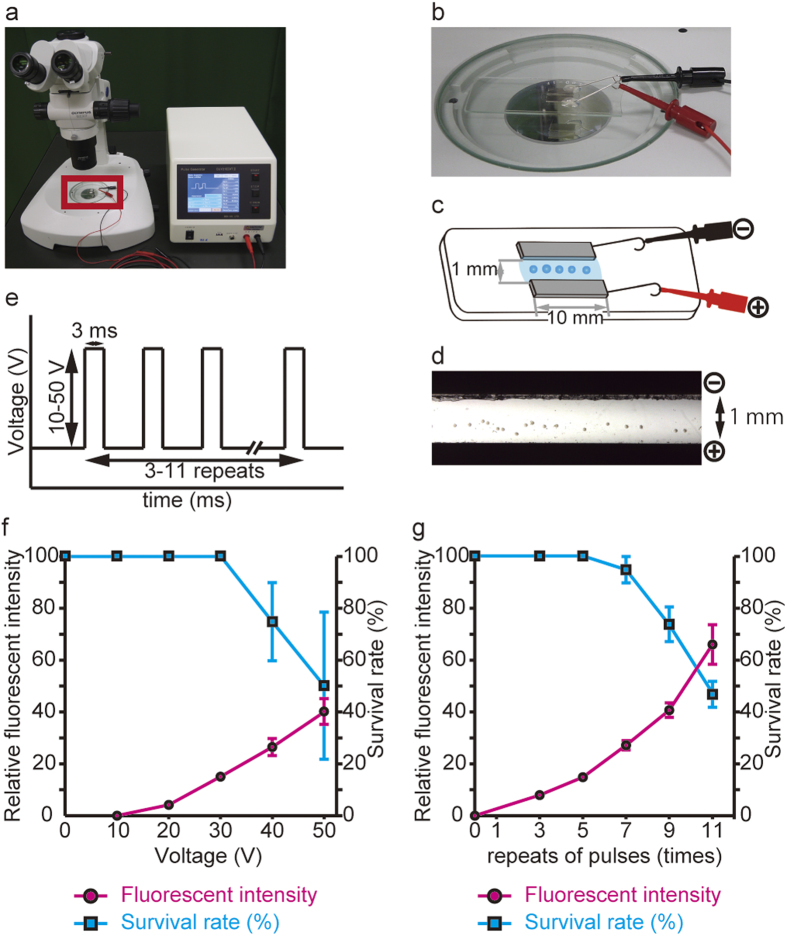
Optimal conditions for the electroporation used to deliver mRNA into fertilized eggs. **(a)** Electroporation set-up used in this study. The right box is the electroporator CUY21 EDIT II (BEX Co. Ltd.), which generates electric pulses, and the left is a stereoscopic microscope for embryo manipulation. Two electrodes were placed on the microscope stage and connected to the electroporator. **(b)** Higher magnification of the red rectangle in (**a**). **(c)** Schematic of the electroporation chamber with customized platinum electrodes (BEX Co. Ltd.). Fertilized mouse eggs were placed in the RNA solution in the gap between the electrodes. **(d)** Microscopic view of the eggs set in the electrode gap. The eggs were manually positioned into a line prior to electroporation. **(e)** Schematic of the electroporation conditions used to introduce RNAs into mouse eggs: three to eleven repeats of a square pulse of 10–50 V; 3-msec pulses with 97-msec intervals were used. **(f)** Fluorescence intensity of mCherry (red circles) and rate of electroporated embryo survival to the blastocyst stage (blue squares) were plotted at various voltages (n = 10). The duration of each pulse and number of pulse repeats were fixed at 3 msec and 5 repeats, respectively. **(g)** The fluorescent intensity of mCherry (red closed circle) and the survival rate of the electroporated embryos to the blastocyst stage (blue squares) were plotted as a function of the number of electroporation repeats (n = 12). The voltage and duration of each pulse were fixed at 30 V and 3 msec, respectively.

A pair of custom-made (BEX, Tokyo, Japan) platinum block electrodes (length: 10 mm, width: 3 mm, height: 0.5 mm, gap: 1 mm) was used (Fig. 1a).

The electrodes, connected to a CUY21EDIT II or CUY21 Vivo-SQ (BEX, Tokyo, Japan), were set under a stereoscopic microscope. The collected eggs cultured in mWM medium were washed with Opti-MEM I three times to remove the serum-containing medium. The eggs were then placed in a line in the electrode gap filled with RNA-containing Opti-MEM I solution (total 5 μl volume), and electroporation was performed. The electroporation conditions were 30 V (3 msec ON + 97 msec OFF) × 7 times in most experiments. After electroporation, the eggs were immediately collected from the electrode chamber and subjected to four washes with M2 medium followed by two washes with mWM medium. The eggs were then cultured in mWM medium at 37 °C and 5% CO2 incstage.

### Fluorescent signal detection and analyses

The signal intensity of the mCherry fluorescence was measured 15 hours after electroporation, using a Nipkow-disc confocal unit CSU-W1 (Yokogawa, Japan) connected to an Axio Observer Z1 inverted microscope (Zeiss, Germany). The fluorescent signal was detected by an EM-CCD camera ImagEM (Hamamatsu Photonics, Japan) and the data were analyzed using the HC image software.

### Genome editing of *Fgf10* and *H2b-mCherry*

The *Cas9* mRNA and gRNAs targeting *Fgf10* or *H2b-mCherry* were introduced into eggs collected from B6D2F1 females by electroporation at E0.5 as described above. For the HDR-mediated knock-in study, 400 ng/μl ssODN was introduced together with 200 ng/μl *Cas9* mRNA and 100 ng/μl gRNA. The sequence of the ssODN was as follows: *H2b-mCherry* (5’- AGTTCATGCGCTTCAAGGTGCACATGGAGGGCTCCGTGAATTCATAACTTCGTATAGCATACATTATACGAAGTTATCGAGGGCGAGGGCCGCCCCTACGAGGGCACCCAGACCGCC -3’). The surviving 2-cell-stage embryos were transferred to the oviducts of pseudopregnant females on the day of the vaginal plug. The mice were dissected at E15-16 (*Fgf10*) or E9 (*H2b-mCherry*), and the embryos were collected.

To investigate CRISPR/Cas9-mediated mutation in the *Fgf10* or *H2b-mCherry* gene, the genomes were prepared from the yolk sac of the embryos. The genomic regions flanking the gRNA target were amplified by PCR using specific primers: *Fgf10* Fwd (5’-CAGCAGGTCTTACCCTTCCA-3’) and Rev (5’-TACAGGGGTTGGGGACATAA-3’), *H2b-mCherry* Fwd (5’-GAGGGCACTAAGGCAGTCAC-3’) or Fwd2 (5’-AAGGGCGAGGAGGATAACAT-3’) and Rev (5’-CCCATGGTCTTCTTCTGCAT-3’). The PCR amplicons of *Fgf10* or *H2b-mCherry* were cloned into the pMD20 (Takara Bio Inc., Shiga, Japan) vector. Ten plasmids from each embryo were isolated, and the genomic region was sequenced. Sequencing was performed using the BigDye terminator Cycle Sequencing Kit ver. 3.1 and ABI 3500 Genetic Analyzer (Applied Biosystems, Foster City, CA).

## Additional Information

**How to cite this article**: Hashimoto, M. and Takemoto, T. Electroporation enables the efficient mRNA delivery into the mouse zygotes and facilitates CRISPR/Cas9-based genome editing. *Sci. Rep.* **5**, 11315; doi: 10.1038/srep11315 (2015).

## Supplementary Material

Supplementary Information

## Figures and Tables

**Figure 2:**
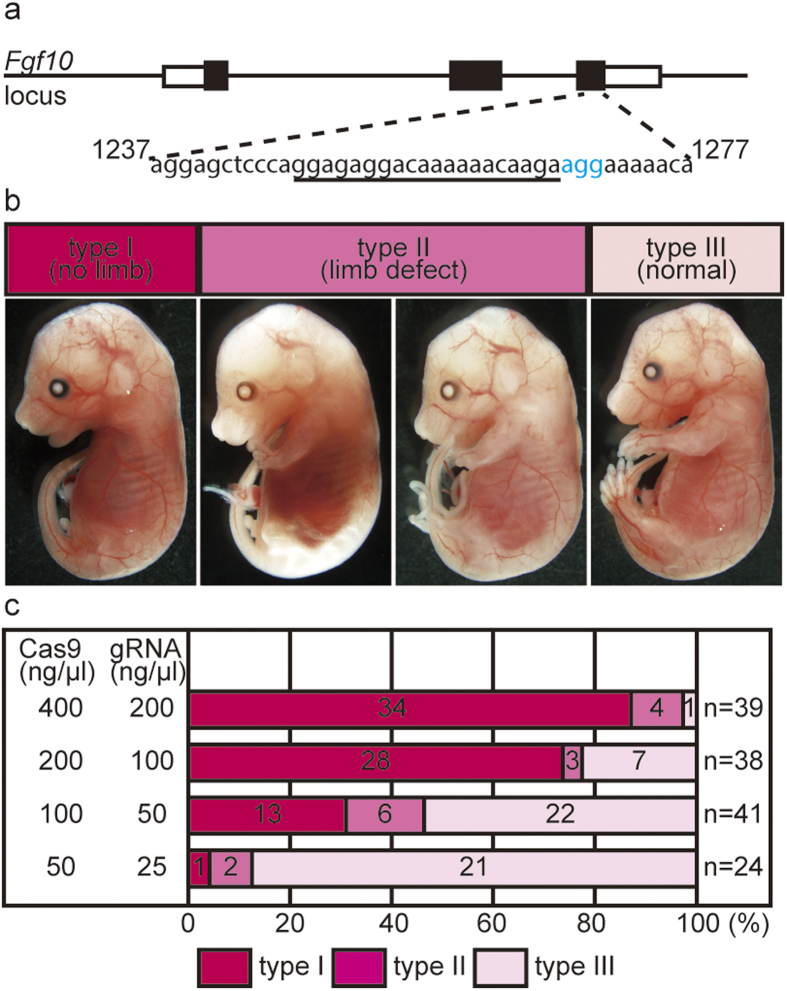
CRISPR/Cas9-mediated genome editing by electroporation. **(a)** Genomic structure of the *Fgf10* locus, which includes the target sequence (underlined) and the protospacer-adjacent motif (PAM) sequence (shown in blue), used in this study. **(b)** Examples of embryos from the three classes that exhibited different limb phenotypes: no limbs (type I), limb defects (type II, left: hindlimb deficiency, right: truncated fore- and hind-limb), and normal (type III). **(c)** Graph summarizing the effects of *Cas9* and gRNA electroporation on limb development. The concentrations of RNAs used in each experiment are shown at left. The numbers in each column are the number of embryos exhibiting the phenotype in each category.

**Figure 3:**
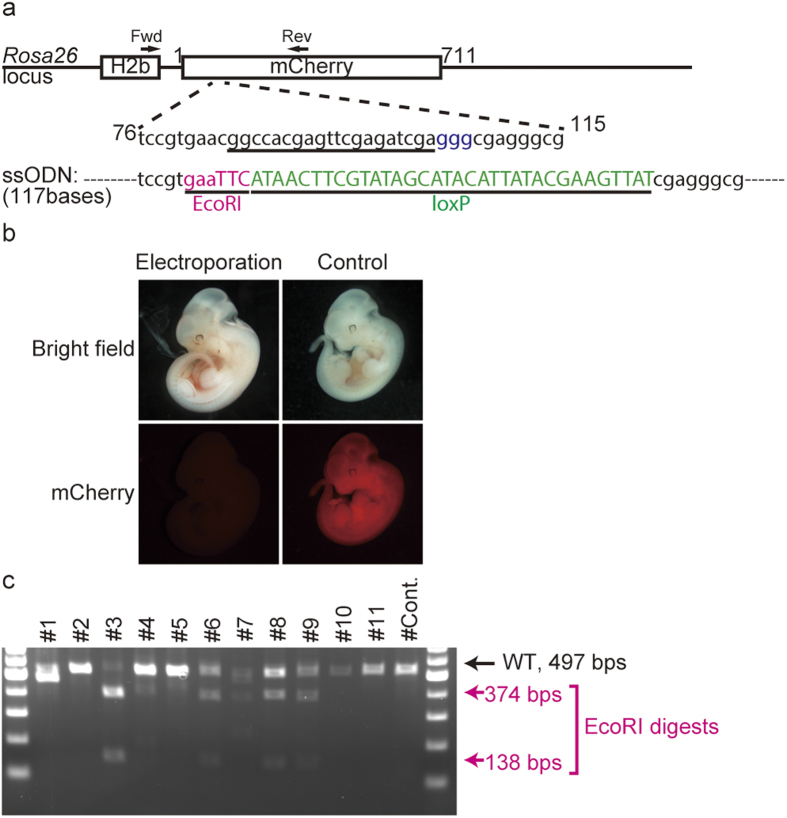
The single-stranded oligodeoxynucleotide (ssODN)-induced generation of HDR-mediated insertions. **(a)** Schematic of the target sequence and the ssODN designed to insert the 37-base loxP sequence (shown in green) and EcoRI recognition site (shown in red). **(b)** Representative embryo electroporated with *Cas9* mRNA, gRNA, and ssODN. The mCherry fluorescence completely disappeared in the electroporated embryos, while control (no electroporation) embryos displayed fluorescent signals. **(c)** RFLP analysis of the collected embryos. The EcoRI-inserted alleles were digested into two bands (138 bps and 374 bps). The intact allele had 497 bps. The digested bands were observed in embryos # 3, 6, 8, and 9. The unexpected bands in #1 and #7 were the target sequence containing a large deletion.
