# Supplementary material for: Electroporation enables the efficient mRNA delivery into the mouse zygotes and facilitates CRISPR/Cas9-based genome editing
Source: Sci Rep. 2015 Jun 11;5:11315. doi: 10.1038/srep11315 (PMC4463957; doi:10.1038/srep11315)
Supplement: Supplementary Information [file srep11315-s1.pdf]

Supplementary Information

Electroporation enables the efficient mRNA delivery into the mouse zygotes and facilitates CRISPR/Cas9-based genome editing.

Masakazu Hashimoto[1,*] and Tatsuya Takemoto[2,*]

[1]Division of Developmental Biology, Graduate School of Medicine, Chiba University, Inohana 1-8-1, Chuo-ku, Chiba, 260-8670, Japan, [2]Division of Embryology, Fujii Memorial Institute of Medical Sciences, The University of Tokushima, 3-18-15 kuramoto-cho, Tokushima 770-8503, Japan

Supplementary Figure S1

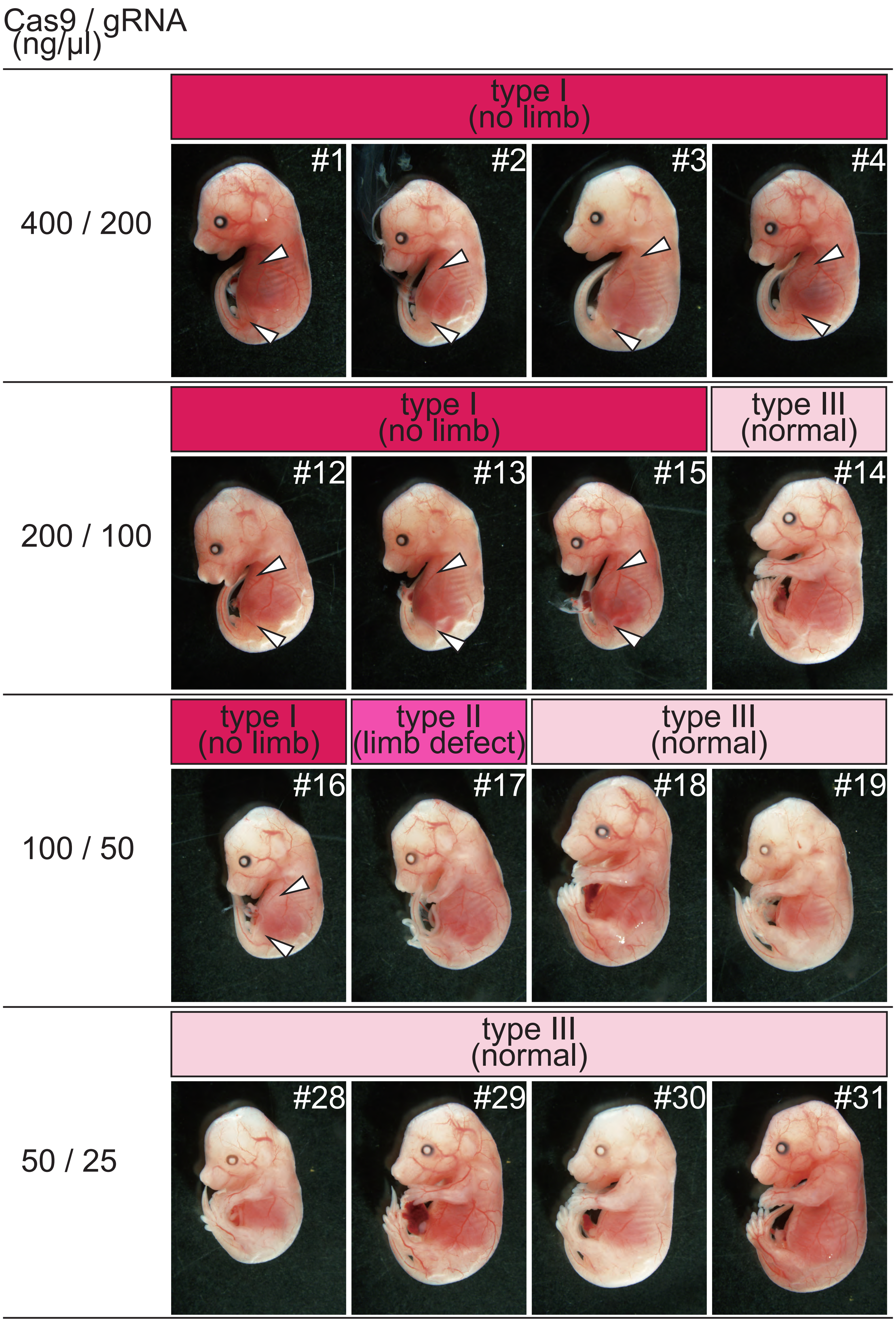

Supplementary Figure S1. Embryos analyzed for their *Fgf10* gene sequence.

The genomic sequence of the *Fgf10* locus was analyzed for four selected embryos generated from eggs electroporated with various concentrations of *Cas9* mRNA and gRNA (Supplementary Table 2). Embryos were categorized into three types (I, II, or III) based on their limb phenotype.

Supplementary Figure S2

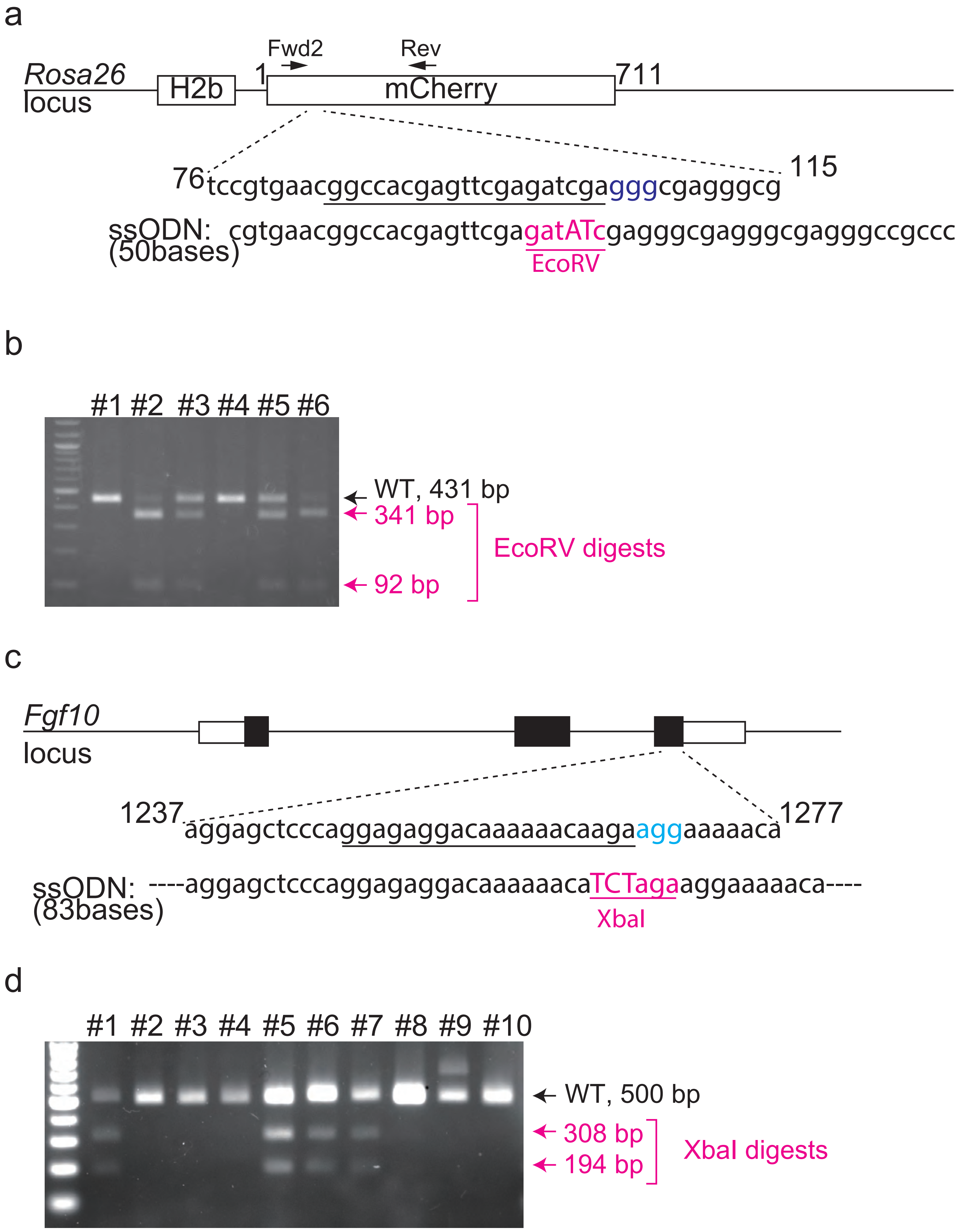

Supplementary Figure 2. HDR-mediated insertion of a restriction enzyme recognition sequence.

(a) Schematic of the target sequence and the ssODN designed to insert the EcoRV recognition site (shown in red) into the *mCherry* locus. The protospacer-adjacent motif (PAM) sequence is shown in blue. Black underline indicates the gRNA recognition sequence (protospacer sequence). (b) RFLP analysis of the collected embryos. EcoRV-inserted alleles were digested into two bands (341 bps and 92 bps). The intact allele had 431 bps. The digested bands were observed in embryos # 2, 3, 5, and 6. The PAM sequence is shown in blue. Black underline indicates the gRNA recognition sequence (Protospacer sequence). (c) Schematic of the target sequence and the ssODN designed to insert the XbaI recognition site (shown in red) into the *Fgf10* locus. (d) RFLP analysis. XbaI-inserted alleles were digested into two bands (308 bps and 194 bps). The two digested bands were observed in embryos #1, 5, 6, and 7.

Supplementary Table S1: Generation of *Fgf10* mutant embyos by *Cas9* mRNA and gRNA electroporation

	Cas9/ sgRNA (ng/µl)	No. electroporation /transferred (%)	No.(%) embryos	No.(%) limb defects
Fgf10	400 / 200	80 / 75 (94)	39 (52)	38 (97)
	200 / 100	63 / 60 (95)	38 (63)	31 (82)
	100 / 50	64 / 60 (94)	43 (72)	19 (46)
	50 / 25	35 / 33 (94)	17 (51)	3 (12)

Supplementary Table S2: Sequence analysis of *Fgf10* mutants

Cas9/gRNA (ng/μl)					
		WT	tgaatggaaaaggagctcccaggagaggacaaaaaacaagaaggaaaaacacctctgctca		
400/200	type I (no limb)	#1	tgaatggaaaaggagctcccaggagaggacaaaaaac---------------ctctgctca	5/10	15 bp deletion
			tgaatggaaaaggag--------------------------aggaaaaacacctctgctca	3/10	26 bp deletion
			tgaatggaaaaggagctcccaggagaggacaaaaa---agaaggaaaaacacctctgctca	2/10	3 bp deletion
		#2	tgaatggaaaaggagctcccaggagaggacaaaaaaca-------------cctctgctca	6/10	13 bp deletion
			tgaatggaaaaggagctcccaggagaggacaaaaa--------------cacctctgctca	4/10	14 bp deletion
		#3	tgaa--------------------------------------ggaaaaacacctctgctca	4/10	38 bp deletion
			tgaatggaaaaggagctcccaggagaggacaaaaaa------ggaaaaacacctctgctca	4/10	6 bp deletion
			tgaatggaaaaggagctcccaggagaggacaaaaa--------------cacctctgctca	1/10	14 bp deletion
			tgaatggaaaaggagctcccaggagaggacaaaaaacaaAgaaggaaaaacacctctgctca	1/10	1 bp insertion
		#4	tgaatggaaaaggag----------aggacaaaaaacaagaaggaaaaacacctctgctca	2/8	10 bp deletion
			tgaatggaaaaggagctcccaggaga---------------aggaaaaacacctctgctca	2/8	15 bp deletion
			tgaatggaaaaggagctcccaggagaggacaaaaa--------------cacctctgctca	2/8	14 bp deletion
			tgaatggaaaaggagctcccaggagaggacaaaaaacaaAgaaggaaaaacacctctgctca	2/8	1 bp insertion
200/100	type I (no limb)	#12	tgaatggaaaaggagctcccaggagag-------------aaggaaaaacacctctgctca	3/10	13 bp deletion
			tgaatggaaaaggagctcccaggagaggacaa----------ggaaaaacacctctgctca	3/10	10 bp deletion
			tgaatggaaaaggagctcccaggagaggacaaaaaaca-------------cctctgctca	2/10	13 bp deletion
			tgaatggaaaaggagctcccaggagaggacaaaaaacaaaaagaaggaaAAAcacctctgctca	1/10	3 bp insertion
			tgaatggaaaaggagctcccaggagaggacaaaaaaAcaagaaggaaaaacacctctgctca	1/10	1 bp insertion
		#13	tgaatggaaaaggagctcccaggagaggacaaaaa-------ggaaaaacacctctgctca	2/8	7 bp deletion
			tgaatggaaaaggagctcccaggagaggacaaaaaaAcaagaaggaaaaacacctctgctca	2/8	1 bp insertion
			tgaatggaaaaggagctcccaggagaggacaaaaaacaaAgaaggaaaaacacctctgctca	2/8	1 bp insertion
			tgaatggaaaaggagctcccaggagaggacaaaa---------------cacctctgctca	1/8	15 bp deletion
			tgaatggaaaaggagctcccaggagaggacaaaaa-caagaaggaaaaacacctctgctca	1/8	1 bp deletion
		#15	tgaatggaaaaggagctcccaggagaggacaaaaaacaaAgaaggaaaaacacctctgctca	5/9	1 bp insertion
			tgaatggaaaaggagctcccaggagaggacaaaaaaca-------------cctctgctca	3/9	13 bp deletion
			---aacacctctgctca	1/9	long deletion
	type III (normal)	#14	tgaatggaaaaggagctcccaggaga---------------aggaaaaacacctctgctca	5/8	15 bp deletion
			tgaatggaaaaggagctcccaggagaggacaaaaaa------ggaaaaacacctctgctca	2/8	6 bp deletion
			tgaatggaaaaggagctcccaggagaggacaaaa---------------cacctctgctca	1/8	15 bp deletion
100/50	type I (no limb)	#16	tgaatggaaaaggagctcccaggagaggacaaaaaaca-------------cctctgctca	5/10	13 bp deletion
			tgaatggaaaaggagctcccaggagaggacaaaaaacaaAgaaggaaaaacacctctgctca	5/10	1 bp insertion
	type II (limb defect)	#17	tgaatggaaaaggagctcccaggagaggacaaaaaaca-------------cctctgctca	8/9	13 bp deletion
			tgaatggaaaaggagctcccaggagaggacaaaaaa---gaaggaaaaacacctctgctca	1/9	3 bp deletion
	type III (normal)	#18	tgaatggaaaaggagctcccaggagaggacaaaaaacaagaaggaaaaacacctctgctca	8/10	wild-type
			TGAATGGAAAAGGAGCTCCCAGGAGAGGACAAAAAAC---------------CTCTGCTCA	2/10	15 bp deletion
		#19	tgaatggaaaaggagctcccaggagaggacaaaaaacaagaaggaaaaacacctctgctca	8/9	wild-type
			tgaatggaaaaggagctcccaggagaggacaaaaaacaagaaggGaaaacacctctgctca	1/9	1 bp mutation
50/25	type III (normal)	#28	tgaatggaaaaggagctcccaggagaggacaaaaaacaagaaggaaaaacacctctgctca	8/10	wild-type
			tgaatggaaaaggagctcccaggagaggacaaaaaacaaAgaaggaaaaacacctctgctca	1/10	2 bp insertion
			tgaatggaaaaggagctcccaggagaggacaaaaaacaagGaggaaaaacacctctgctca	1/10	1 bp mutation
		#29	tgaatggaaaaggagctcccaggagaggacaaaaaacaagaaggaaaaacacctctgctca	7/10	wild-type
			tgaatggaaaaggagctcccaggagaggacaaaaaa---gaaggaaaaacacctctgctca	3/10	3 bp deletion
		#30	tgaatggaaaaggagctcccaggagaggacaaaaaacaagaaggaaaaacacctctgctca	5/10	wild-type
			tgaatggaaaaggagctcccaggagaggacaaaaaGcaagaaggaaaaacacctctgctca	2/10	1 bp mutation
			tgaatggaaaaggagctcccaggagaggacaaaaaacaaCAgaaggaaaaacacctctgctca	2/10	2 bp insertion
			tgaatggaaaaggagctcccaggagaggacaaaaaa--ggaaggaaaaacacctctgctca	1/10	2 bp deletion
		#31	tgaatggaaaaggagctcccaggagaggacaaaaaacaagaaggaaaaacacctctgctca	8/10	wild-type
			tgaatggaaaaggagctcccaggagaggacaaaaaGcaagaaggaaaaacacctctgatca	1/10	1 bp mutation
			tgaatggaaaaggagctcccaggagaggacaaaaaacaagaGggaaaaacacctctgctca	1/10	1 bp mutation

Sequence used as a target is underlined. Blue color indicates the protospacer-adjacent motif (PAM) sequence. Base-substitutions are shown in capitals.

Supplementary Table S3: Sequence analysis of HDR-mediated knock-in embryos

	gtgaacggccacgagttcgagatcgagggcga		
#3	gtgaaTTCATAACTTCGTATAGCATACATTATACGAAGTTATcga	10/10	HDR-mediated knock-in
#6	gtgaaTTCATAACTTCGTATAGCATACATTATACGAAGTTATcga	3/9	HDR-mediated knock-in
	gtgaacggccacgagttcgagatAgagggcga	3/9	1bp insertion
	gtgaacggccacgagttcgagatGTATGCTATACGAAGTTA-------	2/9	unexpected insertion of ssODN
	gtgaacggccacgagttcgaga-cgagggcga	1/9	1bp deletion
#8	gtgaaTTCATAACTTCGTATAGCATACATTATACGAAGTTATcga	5/8	HDR-mediated knock-in
	gtgaacggccacgagttcgaga-cgagggcga	2/8	1bp deletion
	gtgaaTTCATAACTTCGTATAGCATACATTATACGAAGTTATcgagggcga	1/8	unexpected insertion of ssODN
#9	gtgaaTTCATAACTTCGTATAGCATACATTATACGAAGTTATcga	6/8	HDR-mediated knock-in
	gtgaacggccacgagttcgaga-cgagggcga	2/8	1bp deletion

Sequence used as a target is underlined. Blue indicates the PAM sequence. EcoRI site and loxP sequence are shown in red and green, respectively.

Supplementary Table S4: Sequence analysis of HDR-mediated EcoRV site knock-in embryos

	gtgaacggccacgagttcgagatcgagggcga		
#1	gtgaacggccacgagttcgagaTtcgagggcga	4/10	1bp insertion
	gtgaacggccacgagttcgagaGtcgagggcga	3/10	1bp insertion
	gtgaacggccacgagttcgagatAcgagggcga	3/10	1bp insertion
#2	gtgaacggccacgagttcgagatATcgagggcga	10/10	HDR-mediated knock-in
#3	gtgaacggccacgagttcgagat-cgagggcga	5/9	1bp deletion
	gtgaacggccacgagttcgagatATcgagggcga	3/9	HDR-mediated knock-in
	gtgaacggccacgagttcga----cgagggcga	1/9	4bp deletion
#4	gtgaacggccacgagttcgagat-cgagggcga	5/10	1bp deletion
	gtgaacggccacgagttcgagatTcgagggcga	1/10	1bp insertion
	gtgaacggccacgagttcgagatATcGAGGGCGAGGGgagggcga	3/10	unexpected insertion of ssODN
#5	gtgaacggccacgagttcga----cgagggcga	3/10	4bp deletion
	gtgaacggccacgagttcgagatATcgagggcga	6/10	HDR-mediated knock-in
	gtgaacggccacgagttcgagatcgagggcga	1/10	WT
#6	gtgaacggccacgagttcgagatATcgagggcga	1/9	HDR-mediated knock-in
	gtgaacggccacgagttcgagatATcGAGGGgagggcga	1/9	unexpected insertion of ssODN
	gtgaacggccacgagttcgagatATcGAGGGCGAGGGgagggcga	7/9	unexpected insertion of ssODN

Sequence used as a target is underlined. Blue indicates the PAM sequence. EcoRV site is shown in red.

Supplementary Table S5: Sequence analysis of F1 mice generated from mCherry-disrupted F0 mice

F0	F1	ggtgcacatggagggctccgtgaacggccacgagttcgagatcgagggcgaggg	
#171	7 mice	ggtgcacat----------------------------------gagggcgaggg	34bp deletion
#164	2 mice	ggtgcacatggagggctccgtgaacggccacga---------------------	32bp deletion
	1 mice	ggtgcacatggagggctccgtgaacggccacgagttcga----gagggcgaggg	4bp deletion
#165	4 mice	ggtgcacatg---------------------------------gagggcgaggg	33bp deletion

Sequence used as a target is underlined. Blue indicates the PAM sequence.
